# Anaplastic carcinoma of the pancreas producing granulocyte-colony stimulating factor: a case report

**DOI:** 10.1186/1752-1947-2-391

**Published:** 2008-12-17

**Authors:** Atsushi Nakajima, Hirokazu Takahashi, Masahiko Inamori, Yasunobu Abe, Noritoshi Kobayashi, Kensuke Kubota, Shoji Yamanaka

**Affiliations:** 1 Gastroenterology Division, Yokohama City University Hospital, 3-9 Fukuura, Kanazawa-ku Yokohama 236-0004, Japan; 2 Division of Pathology, Yokohama City University Hospital, Yokohama, Japan

## Abstract

**Introduction:**

The granulocyte-colony stimulating factor-producing tumor was first reported in 1977, however, anaplastic pleomorphic type carcinoma of the pancreas producing granulocyte-colony stimulating factor is still rare.

**Case presentation:**

A 63-year-old man was admitted to our hospital with body weight loss (-10 kg during months) and upper abdominal pain from 3 weeks. Abdominal computed tomography demonstrated a pancreatic tumor 10 cm in size and multiple low-density areas in the liver. On admission, the peripheral leukocyte count was elevated to 91,500/mm^3 ^and the serum concentration of granulocyte-colony stimulating factor was 134 pg/mL (normal, < 18.1 pg/mL). Based on liver biopsy findings, the tumor was classified as an anaplastic pleomorphic-type carcinoma. Immunohistochemical staining showed that pancreatic carcinoma cells were positive for granulocyte-colony stimulating factor. The patient developed interstitial pneumonia, probably caused by granulocyte-colony stimulating factor, and died 11 days after admission.

**Conclusion:**

This is a rare case report of anaplastic pleomorphic-type carcinoma of the pancreas producing granulocyte-colony stimulating factor and confirmed by immunohistochemistry.

## Introduction

The granulocyte-colony stimulating factor (G-CSF)-producing tumor was first reported in 1977 by Asano *et al. *in lung cancer [1]. Since that study, further G-CSF-producing lung carcinomas have been reported, but G-CSF-producing pancreatic carcinomas have been very rare [2-7]. Moreover, there have been only a few cases which have reported positive immunostaining for G-CSF in cancer cells [6,7]. We present a case of an anaplastic pancreatic carcinoma with G-CSF production that was confirmed with immunohistochemistry.

## Case presentation

A 63-year-old man was admitted to our hospital with body weight loss (-10 kg during 6 months) and upper abdominal pain. His blood pressure was 123/71 mmHg, pulse was 92 bpm. Physical examination revealed upper left quadrant pain but soft in his abdomen. The tumor was palpable in the upper left abdomen.

Laboratory examination findings were as follows: Peripheral leukocyte count was 91,500/mm^3 ^(87.5% neutrophils, 0% eosinophils, 1.5% lymphocytes, 1% monocytes), hemoglobin was 10.3 g/dL, and platelet count was 38.3 × 10^4^/mm^3^. Serum pancreatic enzymes such as amylase, lipase, and elastase-1 were normal. Serum tumor markers such as sIL-2R (soluble interleukin-2 receptor) and TK (thymidine kinase) were elevated to 2870 U/ml and 15 U/L, respectively, but CEA (Carcinoembryonic Antigen), CA 19-9 (Carbohydrate Antigen 19-9) and NSE (Neuron-specific enolase) were normal. The serum G-CSF was elevated to 134 pg/mL (normal, < 18.1 pg/mL, by enzyme immunoassay).

**Figure 1:**
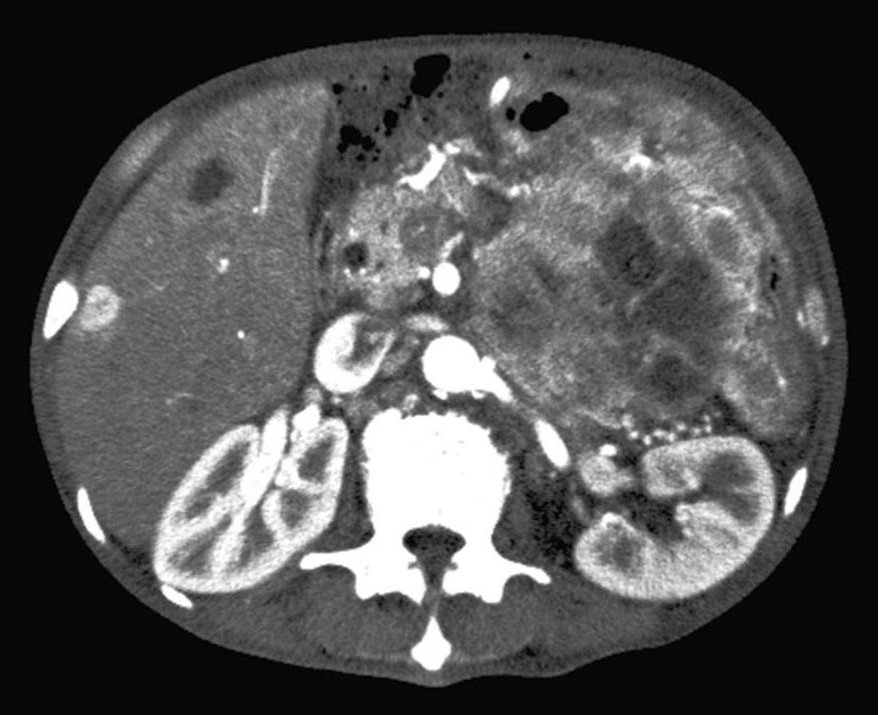
Computed tomography showed an unevenly enhanced mass 10 cm in diameter in the patient's left upper abdomen.

Computed tomography (CT) showed a heterogeneously enhanced mass 10 cm in diameter in the left upper abdomen and multiple low density areas in the liver (see Figure 1). The pancreas could not be detected and it is suggested that the large tumor was originally derived from the pancreas. Magnetic resonance imaging showed a mass of heterogeneous intensity on both T1- and T2-weighted images. Endoscopic examination revealed an extrinsic compression 10 cm in size, at the lesser curve of the body of the stomach. In 2-deoxy-2-[18F]-fluoro-D-glucose positron emission tomography (PET), the maximum standardized uptake value was over 11 at his left upper abdominal lesion. No source of infection was detected. We therefore speculated that this case might be a G-CSF-producing pancreatic carcinoma.

**Figure 2:**
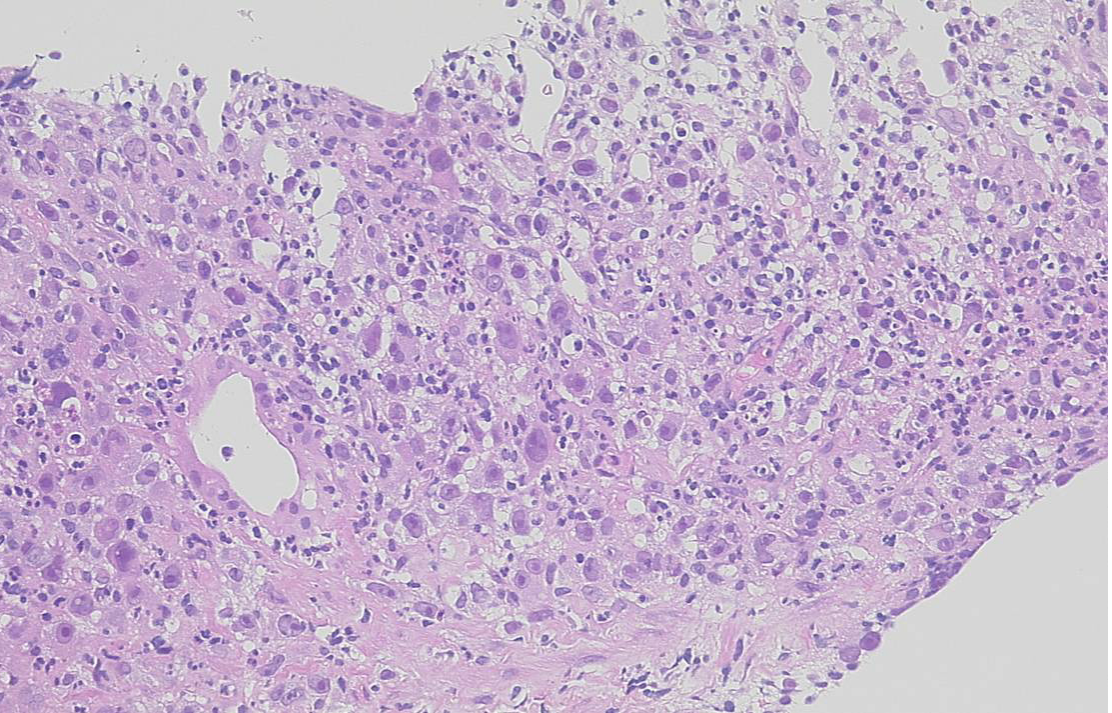
Histopathologic diagnosis of the tumor was an anaplastic pleomorphic-type carcinoma (hematoxylin and eosin stain; ×100).

**Figure 3:**
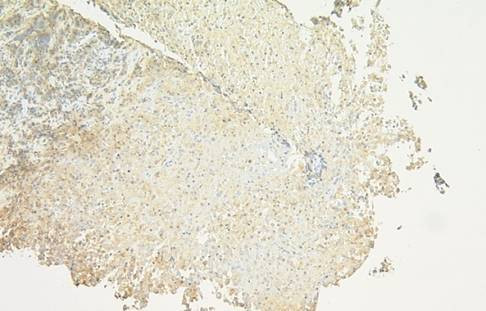
Immunohistochemical staining of formalin-fixed paraffin-embedded liver biopsy material was performed. The pancreatic cancer cells were positive for granulocyte-colony stimulating factor (×40).

Following informed consent, a tumor biopsy of the liver was performed. Histopathologic diagnosis of the tumor was an anaplastic pleomorphic-type carcinoma (see Figure 2). Immunohistochemical staining of formalin-fixed paraffin-embedded liver biopsy material was performed. The pancreatic cancer cells were positive for G-CSF (see Figure 3).

The patient developed interstitial pneumonia, probably caused by G-CSF produced by the carcinoma, and died 11 days after admission.

## Discussion

In 1977, Asano *et al. *[1] reported a case of G-CSF-producing lung cancer. Since then, G-CSF-producing tumors have been reported, however, most cases were of lung cancer origin and G-CSF-producing pancreatic cancer is very rare [2-7].

In the present case, the peripheral leukocyte count was markedly elevated (91,500/mm^3^) on admission, however, no source of infection was detected. Serum G-CSF was elevated to 134 pg/mL. In the liver biopsy material, the histology was anaplastic pleomorphic-type carcinoma and G-CSF was positive on immunohistochemical staining, so we considered that this tumor produced G-CSF. It is uncommon for G-CSF production to be successfully demonstrated with immunohistochemical staining [4,6,7].

Anaplastic carcinoma of the pancreas, also called undifferentiated carcinoma, giant cell carcinoma, pleomorphic large cell carcinoma or sarcomatoid carcinoma, is not common. The incidence of the tumor is only about 2% to 7% of all pancreatic cancers [8-11]. Anaplastic carcinoma has also been rarely identified as a G-CSF-producing tumor [5].

G-CSF-producing tumors are considered to indicate a poor prognosis [2]. In G-CSF-producing lung cancer, large cell tumors and squamous cell tumors are dominant [2]. The 5-year survival rate of large cell tumors is only 14.0% [12]. In addition, Uematsu *et al. *[3] reported that histologic examination of G-CSF-producing carcinomas usually reveals poorly differentiated cells, and moreover, the tumors exhibit rapid growth and are associated with a poor prognosis.

The prognosis of G-CSF-producing carcinomas of the pancreas is also poor. Ohtsubo *et al.*, Kawakami *et al.*, Gotohda *et al.*, Fukushima *et al.*, and our case showed that the survival from tumor detection to death ranged from 11 to 135 days, with a mean of 81.2 days [4-7].

The patient developed interstitial pneumonia and died 11 days after admission. Why did interstitial pneumonia develop? Cases of interstitial pneumonia secondary to treatment with G-CSF have been reported [13]. G-CSF stimulates neutrophils and macrophages. Cytotoxic superoxide from neutrophils and various growth factors from macrophages cause interstitial pneumonia [13]. An increased serum G-CSF level and interstitial pneumonia may be reasons for poor prognosis in patients with G-CSF-producing tumors as in our case.

## Conclusion

This is a rare case report of an anaplastic pleomorphic-type carcinoma of the pancreas producing granulocyte-colony stimulating factor, and confirmed with immunohistochemistry. The clinical characteristics of this disease are still unclear and further detailed studies should be performed.

## Abbreviations

CA19-9: Carbohydrate Antigen 19-9; CEA: Carcinoembryonic Antigen; CT: computed tomography; G-CSF: granulocyte-colony stimulating factor; NSE: Neuron-specific enolase; PET: Positron Emission Tomography; sIL-2R: (soluble interleukin-2 receptor); TK: (thymidine kinase)

## Consent

Written informed consent was obtained from the patient for publication of this case report and any accompanying images. A copy of the written consent is available for review by the Editor-in-Chief of this journal.

## Competing interests

The authors declare that they have no competing interests.

## Authors' contributions

AN: study concept and design, patient care, drafting the manuscript, HT: study concept and design, patient care, drafting the manuscript, MI: study concept and design, patient care, data analysis, literature review, drafting and revising the manuscript, YA: study concept and design, patient care, drafting the manuscript, NK: study concept and design, patient care, drafting the manuscript, literature review, KK: study concept and design, patient care, drafting the manuscript, SY: study concept and design, patient care, drafting the manuscript, literature review. All authors have read and approved the final version of the manuscript.
